# Regulatory Roles of Related Long Non-coding RNAs in the Process of Atherosclerosis

**DOI:** 10.3389/fphys.2020.564604

**Published:** 2020-10-19

**Authors:** Qingyu Meng, Luya Pu, Xizi Luo, Baisen Wang, Fan Li, Bin Liu

**Affiliations:** ^1^Department of Pathogenobiology, The Key Laboratory of Zoonosis, Chinese Ministry of Education, College of Basic Medicine, Jilin University, Changchun, China; ^2^The Key Laboratory for Bionics Engineering, Ministry of Education, Jilin University, Changchun, China; ^3^Engineering Research Center for Medical Biomaterials of Jilin Province, Jilin University, Changchun, China; ^4^Key Laboratory for Health Biomedical Materials of Jilin Province, Jilin University, Changchun, China; ^5^State Key Laboratory of Pathogenesis, Prevention and Treatment of High Incidence Diseases in Central Asia, Xinjiang, China; ^6^Cardiovascular Disease Center, The First Hospital of Jilin University, Changchun, China

**Keywords:** lncRNA, atherosclerosis, vascular cells, lipid metabolism, inflammatory response, angiogenesis

## Abstract

Atherosclerosis (AS) is the main cause of coronary heart disease, cerebral infarction, and peripheral vascular disease, which comprise serious hazards to human health. Atherosclerosis is characterized by the deposition of lipids on the interior walls of blood vessels, causing an inflammatory response of immune cells, endothelial cells, and smooth muscle cells, and a proliferation cascade reaction. Despite years of research, the underlying pathogenesis of AS is not fully defined. Recent advances in our understanding of the molecular mechanisms by which non-coding RNA influences the initiation and progression of AS have shown that long non-coding RNAs (lncRNAs) regulate important stages in the atherosclerotic process. In this review, we summarize current knowledge of lncRNAs, which influence the development of AS. We review the regulatory processes of lncRNAs on core stages of atherosclerotic progression, including lipid metabolism, inflammation, vascular cell proliferation, apoptosis, adhesion and migration, and angiogenesis. A growing body of evidence suggests that lncRNAs have great potential as new therapeutic targets for the treatment of vascular diseases.

## Introduction

Atherosclerosis (AS) is an important risk factor for cardiovascular and cerebrovascular events including myocardial infarction and cerebral infarction (Lu et al., 2015; Sun W. et al., 2018). Continuing research on the pathogenesis of AS has led to the development of several key theories, including the lipid infiltration theory, the injury–response theory, the endothelial function theory, the inflammation theory, and the genetic–environment interaction theory, which reflect the pathogenesis of AS at different levels (Schaftenaar et al., 2016; Kobiyama and Ley, 2018). Atherosclerosis is a slowly progressing disease in which most patients may not show clinical manifestations for years or even decades. The structure of the arterial wall is remodeled during the formation of atherosclerotic plaque. The arterial wall first expands outward, keeping the width of the lumen unchanged. However, if the plaque progresses beyond a certain level, inward progression may occur leading to stenosis. When stenosis exceeds 60% of the lumen diameter, the compensatory expansion ability of the artery may be weakened or even be destroyed, resulting in a series of adverse reactions such as ischemia and thrombosis (Herrero-Fernandez et al., 2019).

Lipid metabolism disorders form the pathological basis of AS, a progressive vascular disease characterized by inflammation, endothelial cell injury, monocyte adhesion, foam cell formation, smooth muscle cell migration from the media to the intima and subsequent proliferation, and atherosclerotic plaque formation (Bäck et al., 2019). Mature atherosclerotic plaques contain large amounts of lipids, foam cells, proliferating smooth muscle cells, and matrix components (Legein et al., 2013). The symptoms of AS depend on the extent of vascular occlusion and the degree of ischemia in the affected organs (Poston and Davies, 1974). Myocardial and cerebral infarctions caused by atherosclerotic involvement are often severely debilitating with high mortality rates (Parthasarathy et al., 2010; Kumarswamy et al., 2014). In recent years, long non-coding RNA molecules (lncRNAs) have been found to play a variety of biological functions in the vascular system, such as promoting apoptosis of endothelial cells (Wang J. et al., 2015), inhibiting the migration of vascular smooth muscle cells (VSMCs; Bell et al., 2014; Zhang et al., 2018), and activating apolipoprotein A1 (APOA1)-mediated macrophage cholesterol efflux (Hu et al., 2014; Pan, 2017). There is abundant showing that lncRNAs are involved in all stages of AS plaque formation, regulating key stages of plaque development, such as lipid metabolism, inflammatory cascade reactions, vascular cell proliferation, apoptosis, adhesion and migration, and angiogenesis (Table 1; Li et al., 2016, 2017; Aryal and Suárez, 2019). Therefore, levels and patterns of lncRNAs in peripheral blood may be clinically useful as markers of AS-related cardiovascular disease and may provide new prevention and diagnostic strategies for AS-related cardiovascular diseases.

**TABLE 1:** Long non-coding RNAs with functional relevance in atherosclerosis.

**Functional classification**	**LncRNA symbol**	**Relation with AS**	**Observed AS characteristics**	**Species**	**References**
Lipid metabolism	DYNLRB2-2	↓	↑ Macrophage cholesterol efflux	h + m	(Hu et al., 2014)
	MeXis	↓	↑ The expression of ABCA1	m	(Sallam et al., 2018)
	RP5-833A20.1	↑	↓ Macrophage cholesterol efflux	h + m	(Hu et al., 2015)
	APOA1-AS	↑	↓ The expression of APOA1	h + m	(Halley et al., 2014)
	NOS3-AS APOA1-AS	↑	↑ TC, LDL-C, oxLDL	h	(Abd-Elmawla et al., 2018)

Inflammatory response	FA2H-2	↑	↑ IL-1β, TNF-α, IL-18, IL-8, IL-6, VCAM-1, MCP-1	h + m	(Guo et al., 2019)
	H19	↑	↑ TNF-α, IL-1β	m	(Han et al., 2018)
	XIAT	↑	↑ IL-1β, IL-6	h	(Hu et al., 2019)
	MALAT1	↓	↓ Inflammatory cells;↑ Autophagy	m/h	(Cremer et al., 2019) (Wang et al., 2019)
	AF131217.1	↓	↓ ICAM-1,VCAM-1	h	(Lu et al., 2019)

Vascular cell proliferation and apoptosis	H19	↑	↑ Proliferation; ↓ Apoptosis (ECs)	h	(Pan, 2017)
	HIF1A-AS1	↑	↑ Apoptosis (damaged ECs)	h	(Wang J. et al., 2015)
	GAS5	↑	↑ Apoptosis (ECs)	h	(Chen et al., 2017)
	HIF1A-AS1	↓	↑ Apoptosis; ↓ Proliferation (VSMCs)	h	(Wang S. et al., 2015)
	p21	↓	↑ Apoptosis; ↓ Proliferation (VSMCs)	h + m	(Wu et al., 2014)
	SMILR	↑	↑ Proliferation (VSMCs)	h	(Ballantyne et al., 2016)

Vascular cell adhesion and migration	AF131217.1	↓	↓ Adhesion of monocytes to ECs	h	(Lu et al., 2019)
	RP11-714G18.1	↓	↓ Adhesion of monocytes to ECs	h	(Zhang et al., 2018)
	MANTIS	↓	↓ Adhesion of monocytes to ECs	h	(Leisegang et al., 2019)
	ANRIL	↑	↑ Adhesion of monocytes to ECs	h	(Samani and Schunkert, 2008) (Holdt et al., 2013)
	ENAST00113	↑	↑ VSMCs proliferation and migration	h	(Yao et al., 2018)
	ENST00000430945	↑	↑ VSMCs proliferation and migration	h	(Cui et al., 2019)
	SENCR	↓	↑ Contractile genes; ↓ Pro-migratory genes	h	(Bell et al., 2014)
	RP11-714G18.1	↓	↓ VSMCs migration; ↓ Angiogenesis	h	(Zhang et al., 2018)

Angiogenesis	TCONS_00024652	↑	↑ HUVECs proliferation and angiogenesis	h	(Halimulati et al., 2018)
	ATB	↑	↑ HMECs viability, migration and angiogenesis	h	(Zhu A.D. et al., 2019)
	HULC	↑	↑ HMECs viability, migration and angiogenesis	h	(Yin et al., 2018b)
	MIAT	↑	↑ HMVECs angiogenesis	h/m	(Yan et al., 2015) (Sun et al., 2019)
	SENCR	↑	↑ HUVECs angiogenesis	h	(Boulberdaa et al., 2016)
	UCA1	↑	↑ HMECs angiogenesis	h	(Yin et al., 2018a)
	MEG3	↓	↓ HMECs migration and angiogenesis;↑ Apoptosis	h + m	(Xu et al., 2020)
	LINC00657	↑	↑ oxLDL-treated HUVECs migration and angiogenesis	h	(Bao et al., 2018)
	H19	↑	↑ Vulnerable plaque formation and intraplaque angiogenesis	m	(Zhang et al., 2017)
	SNHG1	↓	↑ Damaged HUVECs proliferation, migration and angiogenesis	h	(Zhang et al., 2020)

*h, human(cells); m, mouse(cells); +, and; /, or; ABCA1, ATP binding cassette transporter A1; APOA1, apolipoprotein A1; TC, total cholesterol; LDL-C, low-density lipoprotein cholesterol; ox-LDL, oxidized low-density lipoprotein; IL-1β, interleukin-1β; IL-18, interleukin-18; IL-8, interleukin-8; IL-6, interleukin-6; TNF-α, tumor necrosis factor-α; VCAM-1, vascular cell adhesion molecule-1; ICAM-1, intercellular cell adhesion molecule-1; MCP-1, monocyte chemotactic protein-1; ECs, endothelial cells; VSMCs, vascular smooth muscle cells; HUVECs, human umbilical vein endothelial cells; HMECs, human microvascular endothelial cells.*

Research to date shows that complex regulatory mutations may affect the expression of the antisense non-coding RNA (ANRIL) at the INK4 locus, thereby increasing the risk of clinical and subclinical coronary and aortic diseases. Therefore, lncRNA ANRIL is considered to be a risk factor for cardiovascular disease (Johnson et al., 2013). Changes in myocardial infarction-related transcripts (MIAT) that are caused by single nucleotide polymorphisms (SNPs), may be related to the pathogenesis of myocardial infarction, and *in vitro* translation analysis indicates that MIAT did not encode any translation products, indicating that it may be a functional lncRNA (Ishii et al., 2006). Kumarswamy et al. (2014) reported that mitochondrial lncRNA uc022bqs.1 (LIPCAR) is associated with cardiac remodeling and chronic heart failure and may be a potential biomarker. These studies have identified the functional relevance of lncRNAs in cardiovascular disease and helped to delineate the important role played by lncRNAs in AS thrombotic events.

The molecular mechanisms of the lncRNAs involved in the regulation of AS have recently become a new research focus. This review summarizes the core events related to lncRNAs at each stage, including lipid metabolism regulation, inflammation cascade, vascular cell proliferation, apoptosis, adhesion and migration, and angiogenesis. We analyze the role of lncRNAs in the pathogenesis of AS and discuss their potential functions as new therapeutic targets. Long non-coding RNAs may emerge as a relevant target group for early detection and the prognostic monitoring of AS, providing ideas and targets for developing new AS drugs and ultimately realizing the effective prevention and control of AS.

## Brief Introduction of lncRNAs

Non-coding RNA molecules (ncRNAs) comprise a class of RNAs with no protein-coding function that can be divided into three subcategories based on their size: (1) short ncRNAs with 20–50 nucleotides; (2) mid ncRNAs with 50–200 nucleotides; and (3) lncRNAs with greater than 200 nucleotides. Long non-coding RNAs are readily distinguishable from protein coding genes because they lack open reading frames (ORFs) and typical start/stop codons (Cen et al., 2019). Long non-coding RNAs were previously considered to be “transcription noise” because they show a high degree of tissue specificity and are generally less conservative in evolution (Kornfeld and Brüning, 2014). Other studies have shown that lncRNAs are involved in regulating various physiological and pathological processes (Gu et al., 2019), including genetic imprinting, chromatin modification, epigenetic regulation, the cell cycle, and cell differentiation (Qi et al., 2020). Long non-coding RNAs are now known to be important regulators in gene expression networks because lncRNAs can increase or decrease the stability of mRNA in the cytoplasm, activate or inhibit mRNA translation, and post-translational modification processes, and ultimately affect protein expression. In the nucleus, lncRNAs may participate in epigenetic processes and regulate gene expression at the transcriptional and post-transcriptional levels (Kornfeld and Brüning, 2014). The precise molecular mechanisms of lncRNA function remain unclear. Wang and Chang (2011) proposed four functional categories for lncRNAs: signal, bait, guide, and scaffold. In short, an in-depth understanding of the structure and function of lncRNAs will help us explore the regulatory relationship between lncRNAs and the occurrence and development of AS.

## LncRNAs in Lipid Metabolism

The main pathological feature of AS is that macrophages and VSMCs ingest the accumulated lipoprotein in the damaged artery wall, which is finally transformed into foam cells (Chaabane et al., 2014; Zhu et al., 2018; Maguire et al., 2019). This accumulates to form lipid streaks and even lipid plaques, further aggravating the development of AS (Maguire et al., 2019). Activated macrophages enter the blood vessel wall, engulf cholesterol to form foam cells, and then release inflammatory factors, which intensifies the accumulation of additional macrophages. The accumulation of lipids in macrophages and the enhanced inflammatory response are two key factors in the pathogenesis of AS (Nabel and Braunwald, 2012). Studies have shown that the ATP binding cassette transporter A1 (ABCA1) requires APOA1 as a receptor to mediate lipid outflow (Knight, 2004). Hu et al. (2014) found that the highly expressed lncRNA DYNLRB2-2 can upregulate the expression of the G protein coupled receptor 119 (GPR119), and ABCA1 through the glucagon-like polypeptide receptor signaling pathway, activating the APOA1-mediated cholesterol efflux to reduce the cholesterol content in macrophages, and playing an anti-AS function. In addition to lncRNA DYNLRB2-2, lncRNA MeXis plays a key regulatory role in the overload response of macrophages to cholesterol. Sallam et al. found that MeXis can enhance the expression of ABCA1 by regulating the chromatin structure of ABCA1, while suppression of MeXis can accelerate the formation of foam cells and promote AS formation in Apoe^–/–^ mice (Sallam et al., 2018). Using pigs as a model to study fat formation and lipid metabolism, Huang’s team found that 55 lncRNAs were differentially expressed between Laiwu and Large White (Large Yorkshire) pigs (Huang et al., 2018). XLOC_046142, XLOC_004398, and XLOC_015408 were hypothesized to play a key role in the regulation of fat formation and lipid accumulation in pig muscle tissues, while LOC_064871 and XLOC_011001 may play a role in diseases related to lipid metabolism. Reverse cholesterol transport (RCT) maintains lipid metabolism homeostasis and may play a protective role against AS development (Tosheska Trajkovska and Topuzovska, 2017). Studies have found that a site-specific DNA binding protein (NFIA) enhances RCT and inhibits the formation of AS plaques (Song et al., 2010). Hu et al. reported that lncRNA RP5-833A20.1 inhibits the RCT pathway through the RP5-833A20.1/miR-382-5p/NFIA signal transduction pathway and reduces the efflux of excess cholesterol in the body. Thus, the lncRNA RP5-833A20.1 may play an important role in regulating cholesterol homeostasis and inflammation in macrophages (Hu et al., 2015).

Activating apolipoprotein A1 is the main component of high-density lipoprotein (HDL) in plasma. Studies have shown that the lncRNA APOA1-AS recruits histone-modifying enzymes to regulate histone methylation of the APO gene cluster, acting as a negative transcriptional regulator of APOA1, resulting in a reduction in activation markers and an increase of inhibition markers (Halley et al., 2014). In addition to APOA1-AS, other research has revealed that an increase of lncRNA KCNQ1OT1 and lncRNA HIF1A-AS2 in human peripheral blood mononuclear cells (PBMCs) predicts the occurrence of coronary AS and may be useful as a biomarker of coronary artery disease (CAD; Zhang et al., 2019). A recent study showed that patients with systemic lupus erythematosus (SLE) and AS, had higher levels of lncRNA NOS3-AS and lncRNA APOA1-AS expression compared to SLE patients without AS. Systemic lupus erythematosus patients with AS also had higher levels of total cholesterol (TC), low-density lipoprotein cholesterol (LDL-C), and oxidized low-density lipoprotein (ox-LDL) in plasma, suggesting that NOS3-AS and APOA1-AS may be useful as biomarkers of AS in SLE patients (Abd-Elmawla et al., 2018).

## LncRNAs in the Inflammatory Response

Excessive long-term inflammation of the blood vessel wall may lead to increased endothelial cell permeability and enhanced rates of lipid entry, thus exacerbating the development of AS. Inflammatory cytokines and other acute reactants released by endothelial cells during inflammation accelerate endothelial dysfunction (Arnold and Koenig, 2019). A growing body of research indicates that lncRNAs can regulate the balance of inflammatory cytokines within the vascular system. Inappropriate transcriptional activation of innate immunity is a pathological feature of many cardiac and metabolic diseases. Liu et al. (2014) analyzed the lncRNAs in blood and fat cells of experimental subjects exposed to low-dose endotoxins and confirmed that lipopolysaccharides (LPS) regulating lncRNAs originated from fat cells and monocytes. This finding provided new insight into the regulation of inflammatory lncRNAs in cardiac and metabolic diseases at the histological level. Models of endothelial cell inflammation have found that ox-LDL can regulate the expression of certain lncRNAs and play a role in promoting AS (Sun J.J. et al., 2018; Zhu Z. et al., 2019). For example, ox-LDL can down-regulate the expression of lncRNA FA2H-2 in human umbilical vein endothelial cells (HUVECs) to induce inflammation and inhibit autophagic flux, thereby aggravating the development of AS (Guo et al., 2019). Investigating the regulatory effects of lncRNAs on inflammation, Han et al. (2018) found that the expression of lncRNA H19 increased in blood samples of AS patients and cells treated with ox-LDL. Knock down of H19 effectively reduced expression levels of pro-inflammatory factors TNF-α and IL-1β, indicating that H19 can regulate the inflammatory response of cells and provide a new target for AS treatment (Han et al., 2018). Inhibiting the expression of lncRNA XIAT can also reduce ox-LDL-induced inflammation of HUVECs (Hu et al., 2019). In recent years, the role of lncRNA MALAT1 in anti-AS inflammation has attracted attention in the research community. Cremer et al. fed MALAT1-deficient (MALAT1^–/–^) Apoe^–/–^ mice a high-fat diet and found that, compared with Apoe^–/–^ MALAT1^+/+^ mice, Apoe^–/–^ MALAT1^–/–^ mice showed larger plaques and more inflammatory cell infiltration in the vascular wall (Cremer et al., 2019). Subsequent mechanistic studies have shown that the molecular sponge effect of MALAT1 leads to a reduction in miR-503 and anti-inflammatory effects. The molecular sponge effect of MALAT1 was also noted in a study by Wang et al., who found that MALAT1 in HUVECs can adsorb miR-216a-5p and regulate the expression of Beclin-1 to enhance autophagy and inhibit the inflammatory process (Wang et al., 2019), suggesting that MALAT1 may be a potential therapeutic target for AS. Our recent research discovered a new lncRNA, AF131217.1, which is upregulated in HUVECs after laminar shear treatment and can effectively inhibit the expression of vascular cell adhesion molecule-1 (VCAM-1) and intercellular adhesion molecule-1 (ICAM-1; Lu et al., 2019). Continued research and an increasing awareness of lncRNAs will better define the important role that lncRNAs play in inflammation and the development of AS.

## LncRNAs in Vascular Cell Proliferation and Apoptosis

### Endothelial Cells

Oxidative stress can induce apoptosis of vascular endothelial cells, destroy the integrity of monolayer endothelial cells, and increase endothelial permeability, leading to vascular endothelial damage. Abnormal endothelial cell proliferation and apoptosis are important in the initiation and progression of AS (Sun et al., 2013). There is a large body of evidence that lncRNAs are closely related to the proliferation and apoptosis of endothelial cells, highlighting the important role of lncRNAs in the process of AS. Blood levels of lncRNA H19 were found to be higher in AS patients compared to healthy controls. Subsequent *in vitro* experiments confirmed that overexpression of H19 in HUVECs led to an increase in cell proliferation capacity and inhibition of apoptosis (Pan, 2017). In a vascular endothelial cell injury model using palmitic acid (PA), Wang J. et al. (2015) observed that apoptosis of endothelial cells was significantly inhibited in PA treated endothelial cells transfected with the small interfering (si)-lncRNA HIF1A-AS1, suggesting that inhibition of HIF1A-AS1 expression may play a role in vascular endothelial protection.

Research on exosomes in recent years indicates that exosome-derived lncRNAs have an important regulatory effect on the proliferation and apoptosis of endothelial cells. For example, Chen et al. found that vascular endothelial cells undergo apoptosis after ingesting lncRNA GAS5 from THP-1 cell exosomes and that endothelial cell apoptosis is suppressed when GAS5 in exosomes is inhibited (Chen et al., 2017). These results suggest that lncRNAs regulate proliferation and apoptosis of endothelial cells and contribute to the maintenance of endothelial cell function, especially in human endothelial cell models.

### Vascular Smooth Muscle Cells

The normal differentiation of VSMCs requires the fine-tuning of gene expression through microRNAs (Small and Olson, 2011). Due to the close regulatory relationship between lncRNAs and microRNAs, it can be hypothesized that lncRNAs also play a key role in regulating the expression of VSMC differentiation genes, exerting functions related to the phenotype of vascular cells. Other studies have revealed that some lncRNAs play a role in the differentiation and the function of more than one vascular cell type. For example, lncRNA HIF1A-AS1 plays a regulatory role in endothelial cells, and also regulates vascular smooth muscle cells. Studies have found that HIF1A-AS1 can induce aortic VSMC apoptosis by interacting with mammalian chromatin remodeling complex core catalytic subunit related gene 1 (BRG1) and can inhibit abnormal cell proliferation (Wang S. et al., 2015). In addition, lncRNA p21 can inhibit the abnormal proliferation of VSMCs and induce apoptosis. Expression of p21 was significantly down-regulated in the AS plaques of Apoe^–/–^ mice and the lesions of patients with coronary heart disease. Subsequent molecular mechanism studies have shown that p21 can bind to E3 ubiquitin protein ligase (MDM2) to release MDM2 repression of p53 and enhance p53 transcriptional activity (Wu et al., 2014). Activation of p53 can lead to the inhibition of apoptosis and proliferation of VSMCs (Holdt et al., 2016). Conversely, suppression of p53 expression can promote the development of AS (Haupt et al., 1997). The human hyaluronan synthase 2 gene (HAS2) can synthesize hyaluronic acid and promote the proliferation of vascular smooth muscle cells, which is one of the indicators of AS progression. Studies have found that expression of the lncRNA SMILR is increased in human AS plaques. When SMILR in VSMCs is inhibited, the expression of HAS2 may be down-regulated, thereby inhibiting the abnormal proliferation of VSMCs. Thus, the regulation of SMILR may be a new treatment strategy to reduce vascular disease (Ballantyne et al., 2016). Given the growing interest in developing new strategies that directly target diseased cells, the detection of lncRNAs associated with endothelial cells or smooth muscle cells may lead to improved treatment methods.

## LncRNAs in Vascular Cell Adhesion and Migration

### Endothelial Cells

The vascular intima formed by endothelial cells in the cardiovascular system is a “natural container of blood” (Gimbrone, 1987), which may be damaged when stimulated by inflammatory signals. The resulting increase in adhesion molecule expression facilitates the adhesion of monocytes to the damaged endothelial cells and aggravates the progression of AS. We studied the role of lncRNA AF131217.1 in the adhesion of monocytes to endothelial cells. When sh-AF131217.1-3 was used to transfect HUVECs, the number of monocytes adhered to HUVECs increased significantly (Lu et al., 2019), suggesting that AF131217.1 plays an important role in endothelial cell adhesion.

Many lncRNAs can simultaneously conduct different molecular functions. For example, lncRNA RP11-714G18.1 can inhibit VSMC migration and reduce the adhesion of monocytes to endothelial cells (Zhang et al., 2018). According to Leisegang et al., the lncRNA MANTIS can be strictly regulated by the transcription factors KLF2 and KLF4, which may inhibit ICAM-1-mediated adhesion of monocytes to endothelial cells, thereby slowing the progression of AS (Leisegang et al., 2019). The lncRNA ANRIL is associated with cardiovascular disease (Samani and Schunkert, 2008; Holdt et al., 2013). Antisense non-coding RNA has been observed to reduce the apoptosis of human PBMCs and enhance the adhesion of monocytes to endothelial cells through the trans-regulation of target genes, providing molecular mechanisms for the role of ANRIL in promoting AS. Long non-coding RNAs often play an important role in critical periods of AS development, which may be useful for studying the occurrence and development of AS-related cardiovascular diseases.

### Vascular Smooth Muscle Cells

During the pathogenesis of AS, VSMCs migrate from the media to the subintimal space, proliferate abnormally, and accelerate the development of AS lesions. Although some lncRNAs may function as regulators of VSMC migration, their functions and roles in AS have yet to be defined. Yao et al. (2018) observed increased expression of lncRNA ENAST00113 in the blood of AS patients. *In vitro* experiments showed that ENAST00113 can activate the PI3K/Akt/mTOR signaling pathway to promote migration of VSMCs and that down-regulation of ENAST00113 can significantly inhibit proliferation and migration of VSMCs (Yao et al., 2018). Similarly, Cui et al. found that lncRNA ENST00000430945 can promote the proliferation and migration of VSMCs (Cui et al., 2019). These findings indicate that ENAST00113 and ENST00000430945 may play an important role in the migration of VSMCs and suggest that these lncRNAs may be promising therapeutic targets for AS.

LncRNAs that inhibit the migration of VSMCs have also been extensively studied. For example, cytoplasmic lncRNA SENCR transcribed from the 5′ end of the friend leukemia integrated transcription factor 1 (FLI1) gene can inhibit the migration of VSMCs. Likewise, suppressing the expression of SENCR can reduce the expression of smooth muscle contraction genes and increase the expression of migration-associated genes. Zhang et al. (2018) found that lncRNA RP11-714G18.1 can reduce the migration rate of VSMCs and inhibit angiogenesis. SENCR and RP11-714G18.1 may be used as inhibitory molecules for smooth muscle cell migration, to exert certain anti-AS effects (Bell et al., 2014; Zhang et al., 2018). In short, lncRNAs may be useful as regulators of VSMC migration to enhance or inhibit the migration ability of VSMCs, thereby aggravating or alleviating the occurrence and development of AS.

## LncRNAs in Angiogenesis

### Early Stages of AS

Endothelial cell dysfunction is a primary contributor to the pathogenesis of AS, and pathological angiogenesis is a key factor causing endothelial cell dysfunction within the arterial wall (Xiao et al., 2015; Xu et al., 2015). A large number of studies have shown that lncRNAs have an important regulatory role in angiogenesis during the early stages of AS (Halimulati et al., 2018; Yin et al., 2018b; Yu and Wang, 2018; Zhu A.D. et al., 2019). Halimulati et al. (2018) found that lncRNA TCONS_00024652 affects endothelial cell proliferation and angiogenesis by regulating the expression of miR-21. Similarly, miR-21 has been shown to inhibit angiogenesis (Sabatel et al., 2011; Li et al., 2014). Zhu A.D. et al. (2019) studied the functional role of the lncRNA ATB in angiogenesis of human mammary epithelial cells (HMECs) and found that overexpression of ATB enhanced the tube forming ability of HMEC-1 in Matrigel^®^-coated plates while promoting the expression of key angiogenesis factors (VEGF) in pathological angiogenesis (Zachary, 2003; Krishnan et al., 2015). In addition to lncRNA ATB, lncRNA HULC also plays an important role in the angiogenesis of endothelial cells. Studies have found that silencing HULC can significantly reduce the tube formation ability of HMEC-1 and protein expression levels of VEGF and VEGFR2. Subsequent research has found that HULC can adsorb miR-124 and protect myeloid cell leukemia-1 (MCL-1) from degradation by miR-124, thereby accelerating the activation of PI3K/AKT, and JAK/STAT signaling pathways to play a role in promoting angiogenesis (Yin et al., 2018b).

The molecular sponge action of several lncRNAs plays an important role in the molecular mechanisms of angiogenesis. During angiogenesis, the expression of lncRNA MIAT in endothelial cells was significantly up-regulated, and MIAT could up-regulate the expression of VEGF by adsorbing miR-150-5p (Yan et al., 2015). Subsequently, Sun et al. have proposed that MIAT regulates angiogenesis in AS mice by activating the PI3K/Akt signaling pathway (Sun et al., 2019). Thus, MIAT may have important regulatory significance for angiogenesis in the development of AS. The lncRNA SENCR also plays an important role in endothelial cell angiogenesis. After knocking down SENCR in HUVECs, the expression of angiogenic genes (CCL5, CEACAM1, and CX3CL1) was down-regulated (Boulberdaa et al., 2016). This pro-angiogenic effect is also reflected in the lncRNA UCA1. The results of *in vitro* studies by Yin et al. showed that silencing UCA1 can up-regulate miR-195 to inactivate MEK/ERK and mTOR signaling pathways, thereby inhibiting the growth and tube formation ability of HMECs (Yin et al., 2018a). The above-mentioned lncRNAs have shown a pro-angiogenic effect, however, in the pathogenesis of AS, some lncRNAs can inhibit angiogenesis and thus play an anti-AS role. For example, lncRNA MEG3 down-regulates the expression of ICAM-1 by acting as a competing endogenous RNA (ceRNA) of miR-147, thereby inhibiting the growth, migration, and tube formation ability of HMECs and delaying the development of AS (Xu et al., 2020). In short, lncRNAs related to endothelial cell angiogenesis are believed to play an important regulatory role in the occurrence and development of AS.

### The Developmental Stage of AS

The special local environment of atherosclerotic lesions (relative hypoxia, inflammatory aggregation, and oxidative stress) can induce the production of pro-angiogenic factors and stimulate the formation of new blood vessels in AS plaques (Michel et al., 2007; Sluimer and Daemen, 2009; Marsch et al., 2013; Camaré et al., 2017). The formation of new blood vessels increases the supply of nutrients and improves the local hypoxic environment, leading to further plaque development. At the same time, angiogenesis in the plaque intensifies the infiltration of lipids and inflammatory factors that cause intraplaque hemorrhage (IPH), an important factor leading to plaque instability and further AS development (Moreno et al., 2006; Michel et al., 2014). Therefore, exploring the process of angiogenesis in plaques and mechanisms that promote AS development may aid in the discovery of new anti-AS therapeutic targets. Bao et al. (2018) found that in HUVECs treated with ox-LDL, the highly expressed lncRNA LINC00657 can attenuate the inhibitory effect of miR-590-3p on hypoxia-inducible factor-1α (HIF-1α) and upregulate VEGF, MMP-2, and MMP-9 expression to promote endothelial cell proliferation, migration, and angiogenesis. Long non-coding RNAs H19 is closely related to the occurrence and development of CAD in the general population (Zhang et al., 2017), and H19 levels are higher in the peripheral blood of AS patients (Gao et al., 2015). Yang et al. found that H19 up-regulated the expression of MMP-2, VEGF, and p53 in Apoe^–/–^ mice and down-regulated expression of the tissue inhibitor of metalloproteinases-1 (TIMP-1), thus promoting angiogenesis in plaques of AS mice (Yang et al., 2019). A recent study found that the lncRNA SNHG1/miR-196a/MAPK6 axis is involved in regulating the proliferation, migration, and angiogenesis of HUVECs induced by TNF-α, indicating that SNHG1 may be a potential molecular marker of AS (Zhang et al., 2020). There is a large amount of evidence indicating that angiogenesis in plaques may be a core event in the progression of AS, and further in-depth study of relevant lncRNAs in the future will revolutionize our understanding of the etiology of AS.

## Conclusion and Perspective

**FIGURE 1:**
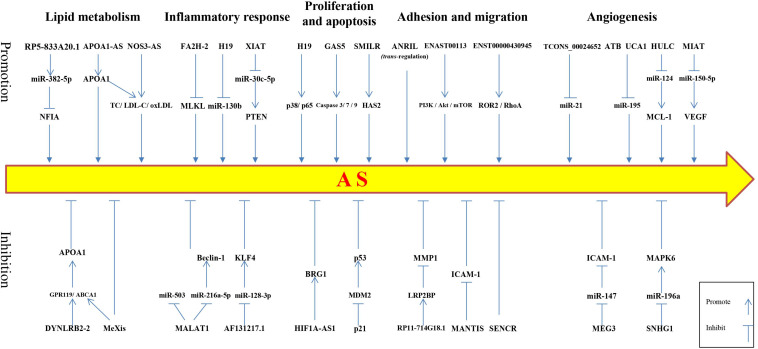
Schematic of long non-coding RNAs regulating atherosclerotic processes. The upper row depicts lncRNAs that promote the development of atherosclerosis, whereas, the lower row shows lncRNAs that inhibit the development of atherosclerosis. NFIA, nuclear factor I-A; APOA1, apolipoprotein A1; TC, total cholesterol; LDL-C, low-density lipoprotein cholesterol; ox-LDL, oxidized low-density lipoprotein; MLKL, mixed lineage kinase domain-like protein; PTEN, phosphatase and tensin homolog deleted on chromosome 10; HAS2, hyaluronan synthase 2; ROR2, receptor tyrosine kinase-like orphan receptor 2; RhoA, ras homolog gene family member A; MCL-1, myeloid cell leukemia-1; VEGF, vascular endothelial growth factor; GPR119, G protein coupled receptor 119; ABCA1, ATP binding cassette transporter A1; KLF4, Kruppel-like factor 4; BRG1, brahma-related gene 1 protein; MDM2, mouse double minute 2; ICAM-1, intercellular cell adhesion molecule-1; MAPK6, mitogen-activated protein kinase 6.

Recent evidence indicates that a large portion of the mammalian genome is transcribed into ncRNA. Long non-coding RNAs are important epigenetic regulators involved in gene expression. Long non-coding RNAs play different physiological or pathological roles under different conditions, regulating the functions of cells and tissues in the body. Growing evidence suggests that lncRNAs play an important role and may become effective targets for intervention in AS-related cardiovascular diseases (Figure 1). In recent years, research on lncRNAs has greatly changed our views of disease etiology, This discovery provides hope that the difficult problems surrounding human cardiovascular diseases can be avoided and prevented. For example, rapamycin (RPM) is often used as a drug coating for drug-eluting stents (DES) because it can inhibit the growth of smooth muscle cells. However, RPM also inhibits the proliferation and migration of vascular endothelial cells and damages the endothelium during DES implantation. Therefore, it is important to develop strategies to protect vascular endothelial cells after DES implantation. Interestingly, overexpression of lncRNA SENCR after RPM treatment can significantly reduce the inhibitory effects of RPM on HUVEC proliferation, migration, and cell cycle progression (Sun H. et al., 2018). Thus, SENCR may be used as a new combination agent to overcome the limitations of RPM in DES implantation. Integrating lncRNAs into traditional treatment strategies has great potential and should be an important aspect of future disease research. Although current research on lncRNAs gives us great expectations for the prevention and treatment of diseases, many important problems remain unresolved. For example, expression levels for most lncRNAs in the body are very low, which complicates the reliability and repeatability of large-scale lncRNA research. Normally, protein-coding transcripts are transported from the nucleus to the cytoplasm and combined with ribosomes to translate proteins; however, most ncRNA remains in the nucleus, making structural and functional studies difficult. Moreover, recent studies have suggested that some lncRNAs are contained in fluid exosomes and that these exosome-contained lncRNAs may play an important role in the development and diagnosis of a variety of human diseases (Gezer et al., 2014; Işın et al., 2015).

Several key issues that must be addressed to fully realize the therapeutic potential of lncRNAs in AS include: (1) the exact biological role of lncRNAs in AS and AS-related cardiovascular diseases; (2) the ability to develop drugs that mimic lncRNA functions and simulate ncRNA *in vivo*; (3) the safety and adverse reactions of lncRNA treatment; and (4) developing optimal animal models for preclinical studies (Devaux et al., 2015). The cost of discovering clinically useful lncRNAs is very high due to the fact that it involves a large amount of gene sequencing and requires a high analytical capacity to accurately predict biological functions. Subsequently, many more *in vitro* and *in vivo* experiments will be needed to further verify previous experiments. The functions of newly discovered lncRNAs are normally inferred indirectly from the known function of their target mRNAs because little functional information can be obtained from the primary sequence of lncRNAs (Li et al., 2016). A growing body of evidence indicates that lncRNAs play a vital regulatory role in various biological processes. A more complete understanding and recognition of their role in the field of human diseases, especially cardiovascular diseases, will aid in the development of novel diagnostic and therapeutic methods. Despite the therapeutic promise of lncRNAs, there are many problems related to integrating lncRNAs into pre-existing miRNA–mRNA–protein regulatory networks, or mRNA–protein regulatory networks remain unresolved. Although studies have found that hundreds of lncRNAs are associated with cardiovascular diseases, the role of these lncRNAs in the diagnosis, prognosis, and treatment of diseases requires further verification.

## Author Contributions

QM and LP contributed to the data curation and writing of the original draft. XL contributed to the visualization and validation. BWcontributed to the software and investigation. FL contributed to the conceptualization, funding acquisition, and supervision. BL contributed to the supervision, writing, reviewing, and editing. All authors contributed to the article and approved the submitted version.

## Conflict of Interest

The authors declare that the research was conducted in the absence of any commercial or financial relationships that could be construed as a potential conflict of interest.
