# Supplementary figures and images for: Regulatory Roles of Related Long Non-coding RNAs in the Process of Atherosclerosis
Source: Front Physiol. 2020 Oct 19;11:564604. doi: 10.3389/fphys.2020.564604 (PMC7604474; doi:10.3389/fphys.2020.564604)

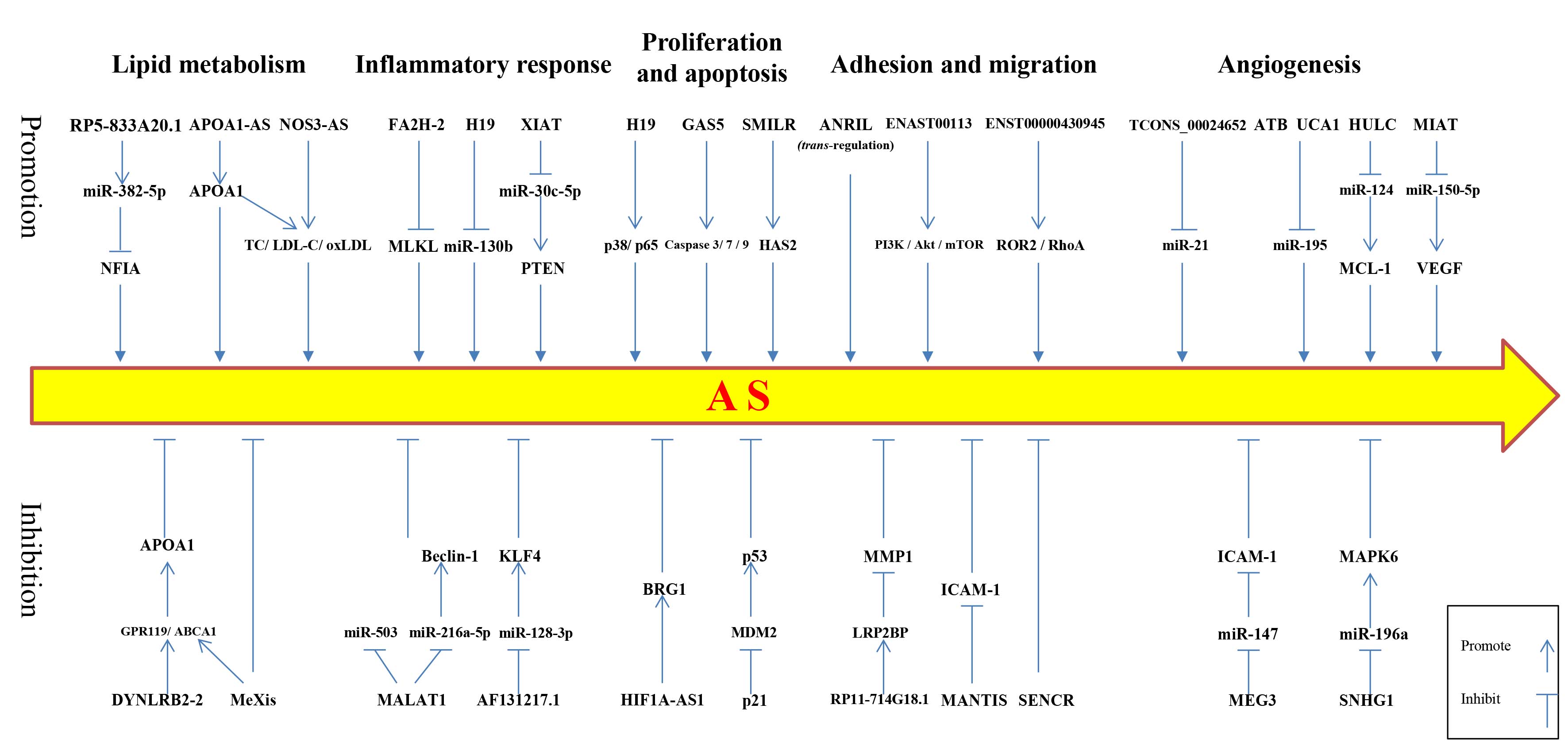

Supplement: Supplementary file 1 [file Figure_1.JPEG]
