# Supplementary material for: Regulatory Roles of Related Long Non-coding RNAs in the Process of Atherosclerosis
Source: Front Physiol. 2020 Oct 19;11:564604. doi: 10.3389/fphys.2020.564604 (PMC7604474; doi:10.3389/fphys.2020.564604)
Supplement: Supplementary file 2 [file Table_1.DOCX]

**Table 1**. Long non-coding RNAs with functional relevance in atherosclerosis.

| **Functional classification** | **LncRNA symbol** | **Relation with AS** | **Observed AS characteristics** | **Species** | **Refs** |
| --- | --- | --- | --- | --- | --- |
| Lipid metabolism | DYNLRB2-2 | ↓ | ↑ Macrophage cholesterol efflux | h+m | [15] |
|  | MeXis | ↓ | ↑ The expression of ABCA1 | m | [31] |
|  | RP5-833A20.1 | ↑ | ↓ Macrophage cholesterol efflux | h+m | [35] |
|  | APOA1-AS | ↑ | ↓ The expression of APOA1 | h+m | [36] |
|  | NOS3-AS APOA1-AS | ↑ | ↑ TC, LDL-C, oxLDL | h | [38] |
|  |  |  |  |  |  |
| Inflammatory response | FA2H-2 | ↑ | ↑ IL-1β, TNF-α, IL-18, IL-8, IL-6, VCAM-1, MCP-1 | h+m | [43] |
|  | H19 | ↑ | ↑ TNF-α, IL-1β | m | [44] |
|  | XIAT | ↑ | ↑ IL-1β, IL-6 | h | [45] |
|  | MALAT1 | ↓ | ↓ Inflammatory cells;↑ Autophagy | m/h | [46][47] |
|  | AF131217.1 | ↓ | ↓ ICAM-1,VCAM-1 | h | [48] |
|  |  |  |  |  |  |
| Vascular cell proliferation and apoptosis | H19 | ↑ | ↑ Proliferation; ↓ Apoptosis（ECs ） | h | [14] |
|  | HIF1A-AS1 | ↑ | ↑ Apoptosis（damaged ECs ） | h | [11] |
|  | GAS5 | ↑ | ↑ Apoptosis（ECs ） | h | [50] |
|  | HIF1A-AS1 | ↓ | ↑ Apoptosis;↓ Proliferation （VSMCs） | h | [52] |
|  | p21 | ↓ | ↑ Apoptosis;↓ Proliferation （VSMCs） | h+m | [53] |
|  | SMILR | ↑ | ↑ Proliferation（VSMCs） | h | [56] |
|  |  |  |  |  |  |
| Vascular cell adhesion and migration | AF131217.1 | ↓ | ↓Adhesion of monocytes to ECs | h | [48] |
|  | RP11-714G18.1 | ↓ | ↓Adhesion of monocytes to ECs | h | [13] |
|  | MANTIS | ↓ | ↓Adhesion of monocytes to ECs | h | [58] |
|  | ANRIL | ↑ | ↑Adhesion of monocytes to ECs | h | [59][60] |
|  | ENAST00113 | ↑ | ↑ VSMCs proliferation and migration | h | [61] |
|  | ENST00000430945 | ↑ | ↑ VSMCs proliferation and migration | h | [62] |
|  | SENCR | ↓ | ↑ Contractile genes;↓ Pro-migratory genes | h | [12] |
|  | RP11-714G18.1 | ↓ | ↓ VSMCs migration; ↓Angiogenesis | h | [13] |
|  |  |  |  |  |  |
| Angiogenesis | TCONS_00024652 | ↑ | ↑ HUVECs proliferation and angiogenesis | h | [66] |
|  | ATB | ↑ | ↑ HMECs viability, migration and angiogenesis | h | [67] |
|  | HULC | ↑ | ↑ HMECs viability, migration and angiogenesis | h | [68] |
|  | MIAT | ↑ | ↑ HMVECs angiogenesis | h/m | [73][74] |
|  | SENCR | ↑ | ↑ HUVECs angiogenesis | h | [75] |
|  | UCA1 | ↑ | ↑ HMECs angiogenesis | h | [76] |
|  | MEG3 | ↓ | ↓ HMECs migration and angiogenesis;↑ Apoptosis | h+m | [77] |
|  | LINC00657 | ↑ | ↑ oxLDL-treated HUVECs migration and angiogenesis | h | [84] |
|  | H19 | ↑ | ↑ Vulnerable plaque formation and intraplaque angiogenesis | m | [85] |
|  | SNHG1 | ↓ | ↑ Damaged HUVECs proliferation, migration and angiogenesis | h | [88] |

Note: h: human(cells); m: mouse(cells); +: and; /: or; ABCA1: ATP binding cassette transporter A1; APOA1: apolipoprotein A1; TC: total cholesterol; LDL-C: low-density lipoprotein cholesterol; ox-LDL: oxidized low-density lipoprotein; IL-1β: interleukin-1β; IL-18: interleukin-18; IL-8: interleukin-8; IL-6: interleukin-6; TNF-α: tumor necrosis factor-α; VCAM-1: vascular cell adhesion molecule-1; ICAM-1: intercellular cell adhesion molecule-1; MCP-1: monocyte chemotactic protein-1; ECs: endothelial cells; VSMCs: vascular smooth muscle cells; HUVECs: human umbilical vein endothelial cells; HMECs: human microvascular endothelial cells.
